# Quantitative proteomic analysis of age-related subventricular zone proteins associated with neurodegenerative disease

**DOI:** 10.1038/srep37443

**Published:** 2016-11-18

**Authors:** Xianli Wang, Chuanming Dong, Lixin Sun, Liang Zhu, Chenxi Sun, Rongjie Ma, ke Ning, Bing Lu, Jinfu Zhang, Jun Xu

**Affiliations:** 1 East Hospital, Tongji University School of Medicine, Shanghai, China; 2 Department of Anatomy, Nantong University, Nantong, Jiangsu, China; 3 Department of Neuroscience, Sheffield Institute for Translational Neuroscience (SITraN), University of Sheffield, Sheffield, UK; 4 Department of Urology, Shanghai Tongren Hospital, Shanghai Jiao Tong University School of Medicine, Shanghai, China

## Abstract

Aging is characterized by a progressive decline in the function of adult tissues which can lead to neurodegenerative disorders. However, little is known about the correlation between protein changes in the subventricular zone (SVZ) and neurodegenerative diseases with age. In the present study, neural stem cells (NSCs) were derived from the SVZ on postnatal 7 d, 1 m, and 12 m-old mice. With age, NSCs exhibited increased SA-β-gal activity and decreased proliferation and pool size in the SVZ zone, and were associated with elevated inflammatory chemokines and cytokines. Furthermore, quantitative proteomics and ingenuity pathway analysis were used to evaluate the significant age-related alterations in proteins and their functions. Some downregulated proteins such as DPYSL2, TPI1, ALDH, and UCHL1 were found to play critical roles in the neurological disease and PSMA1, PSMA3, PSMC2, PSMD11, and UCHL1 in protein homeostasis. Taken together, we have provided valuable insight into the cellular and molecular processes that underlie aging-associated declines in SVZ neurogenesis for the early detection of differences in gene expression and the potential risk of neurological disease, which is beneficial in the prevention of the diseases.

Aging is a process characterized by the progressive decline in the physiology and function of adult tissues^1^,^2^. Studies have shown that the neurogenesis declined rapidly in the human brain with increased age. As a result, the elderly individuals exhibit deteriorated cognitive function^3^ and are largely susceptibility to neurodegenerative diseases such as Parkinson’s and Alzheimer’s diseases^4^. This may be attributed to the degeneration of self-renewal and multi-differentiation potential of neural stem cells (NSCs) associated with NSC aging^5^.

Adult NSCs reside in the subgranular zone (SGZ) of the hippocampal dentate gyrus and the subventricular zone (SVZ) of the lateral ventricle^6^,^7^. Adult NSCs serve as the nascent fountain critical for brain homeostasis. However, the number of NSCs significantly decreases with age, correlating with a functional decline and a gradual loss of olfactory function^8^,^9^. When NSCs are inclined towards aging, some aging-related neurodegenerative diseases begin to occur^10^. The pathological process in Parkinson’s disease (PD) involves the degeneration of the dopaminergic neurons in the substantia nigra pars compacta, which leads to a decrease in the striatal dopamine levels and also causes movement disorder^11^. SVZ is localized in the proximity of the striatum. The endogenous NSCs in the SVZ can migrate into the striatum and differentiate into dopaminergic neurons. With age, the proliferation of endogenous NSCs is decreased, and hence, the number of dopaminergic neurons in the striatum is reduced^12^.

Accumulating evidence showed that the Alzheimer’s disease (AD) influences the SVZ cell proliferation^13^. A recent study indicated a significant nine-fold decrease of Musashi 1-positive progenitor cells in the SVZ of patients with Alzheimer’s disease^14^. The neurogenic capacity of the SVZ is the only source of long-term self-renewable and multipotent NSCs in the adult rodent brain, and thus, is crucial for AD. On the other hand, the SGZ contains only independent neuronal and glial progenitors with limited self-renewal capacity. Therefore, it has been proposed that SVZ NSCs could migrate into the hippocampus, acting as a source of NPCs for the SGZ^15^.

Hitherto, a proteomic study correlating the age-dependent NSC alterations and neurodegenerative diseases is not reported. Therefore, a proteomic analysis would be beneficial for the early detection of the differences in the gene expression and the potential risk of illness, thereby, preventing the neurodegenerative diseases.

A recent study demonstrated that impairment of neurogenesis in the SGZ begins at 9 m in male 3 Tg-AD mice^16^, whereas the SVZ impairment begins as early as 2–3 m^17^. Moreover, the SVZ NSCs reside within the walls of the lateral ventricle. These NSCs from the sequestered parts of the human brain can be endoscopically harvested, expanded *in vitro*, and differentiated into both neuronal and glial progenitors. Thus, the SVZ was selected for the present study.

Herein, we utilized the quantitative proteomic analysis to generate protein expression profiles of the SVZ from the mice at various ages. A comparison of the protein profiles of these groups revealed a number of differentially expressed proteins that are known to play critical roles in the protein homeostasis and neurodegenerative diseases. This information may provide valuable insight into the cellular and molecular processes underlying the aging-associated decline in SVZ neurogenesis.

## Material and Methods

The animal experiment was approved and conducted according to the regulations set by the Animal Use and Care Committee of Tongji University. And all experiments were carried out in accordance with the manufacturer’s instructions.

### Neurosphere culture

Primary NSC culture was derived from postnatal day 7(7d), one month (1 m) and 12 month (12 m) mice. All mice were housed according to Animal care and all experiment procedures approved by the Animal Committee of the school (TJmed-010-10) and the protocol was approved by the Institutional Animal Care and Use Committee of the Tongji University (Shanghai, China). The SVZ was isolated and enzymatically dissociated in Hank’s balanced saline solution buffer (HBSS) (Invitrogen, USA) containing 1 mg/ml trypsin (Invitrogen) at 37 °C for 10 minutes followed by 5 min centrifugation at 350 g upon trypsin inhibition (Invitrogen). The isolated cells were washed with HBSS and resuspended in DMEM/F12 medium (Invitrogen) with 2% B27 (Invitrogen), 20 ng/ml EGF (R&D Systems, USA), 20 ng/ml bFGF (R&D Systems), 2 mM glutamax (Invitrogen). Neurospheres formed over 3 days of incubation with 5% CO_2_ at 37 °C. Subculturing was done every 3–4 days. Experiments were performed with cultured cells between passages 3 and 7.

### Senescence-associated-β-galactosidase assay

Cellular senescence was determined by SA-β-gal staining^18^. Staining was performed using SA-β-gal staining Kit (Genmed Scientifics INC) according to manufacturer’s guidelines with DAPI as counterstaining. Positive staining was evaluated after 12–16 hr incubation at 37 °C in a CO_2_-free atmosphere. The blue stained cells from 10 different fields were counted with results presented as a percentage of positive cells.

### Cell proliferation assays

After trypsinizing neurospheres, single cell suspensions were prepared in DMEM/F12 medium containing EGF and bFGF, and were seeded into 96-well plates (Corning Incorporated, USA) at a density of 10^3^ cells per ml. After 7-days, colonies of a diameter >30 μm were scored.

### Immunocytochemistry

Cells grown on coverslips were fixed in 4% paraformaldehyde for 15 min at room temperature, permeabilized with 0.3% Triton X-100 in PBS for 10 min, and blocked in 3% normal donkey serum in PBS for 2 h at room temperature. Primary antibodies were diluted in 3% normal donkey serum in PBS and applied at 4 °C overnight. The primary antibodies used in these experiments were as follow: Nestin (Abcam; 1:3000), Sox2 (Santa Cruz; 1:500), after rinsing in PBS three times and incubating for 2 h with CF488 and CF543 (Biotium; 1:1000), coverslips were washed three times, cell nuclei were stained with DAPI. Images were acquired on a Leica TCS SP2 confocal fluorescence microscope.

### Immunohistochemistry

Mice were anesthetized and cardiac perfused through the heart with 4% paraformaldehyde. Brains were dissected out, post-fixed and stored in 30% sucrose. Brains were frozen in Tissue-Tek optimal cutting temperature compound (O.C.T.) (VWR, Richmond, IL) and coronally sectioned at a 25 μm interval. Tissue sections were blocked with 3% normal donkey serum (NDS, Jackson) in PBS for 30 minutes and then incubated with Nestin (Abcam; 1:3000) at 4 °C overnight followed by CF488 (Biotium; 1:1000) for 1 hour. DAPI was used as counter staining. Images were acquired and analyzed on a Leica TCS SP2 confocal fluorescence microscope.

### Quantitative RT-PCR

For QRT-PCR, total RNA was reverse-transcribed using SuperScript III (Invitrogen). All reactions were performed using SYBR^®^ Green PCR Core reagents (Applied Biosystems). Primers were designed using Primer Express software (Applied Biosystems) and experimentally validated. The sequences for primers used are as follows: GAPDH 5′ GGTGAAGGTCGGTGTGAACG-3′, 5′ CTCGCTCCTGGAAGATGGTG-3′; PSMD11 5′GAGCCCAGTCTCTACTCAGCA-3′, 5′TCTTGAATGTCACGTTTCACGAT-3′; ALDH2 5′GAAATGTCTCCGCTATTACGCT-3′, 5′GCGGGAAGTTCCACGGAAT-3′; TPI1 5′ GAGAGAGCCGTGCGTTTGTA-3′, 5′ CCCCAACGAAGAACTTCCTGG-3′. The expression of each gene was defined from the threshold cycle (C_t_), and relative expression levels were calculated by using the ∆∆CT method after normalization with reference to expression of the housekeeping gene GAPDH. Results are means from three individual experiments.

### Western blot analysis

Cultured NSCs were washed with ice-cold PBS, lysed with 2 × SDS lysis buffer. Protein concentrations were determined by the BCA protein assay kit (Pierce). Proteins were separated on 8% SDS-polyacrylamide gels (Bio-rad) and transferred to a PVDF membrane (Millipore). Membranes were blocked in 5% non-fat milk powder in TBS-T (0.1% Tween-20 in TBS), and incubated with primary antibodies overnight at 4 °C. After washed in TBS-T, membranes were incubated with HRP conjugated secondary antibodies (Invitrogen) for 1 h at room temperature with signals detected using ECL Super Signal (Pierce, Rockford, IL, USA). Quantifications were done using ImageQuant. The primary antibodies used include rabbit anti-PSMD11 (Abcam) and mouse anti-βactin (Sigma).

### Multiplex quantification of cytokines and chemokine

Concentrations of cytokine/chemokine in 7d, 1 m and 12 m were NSCs were quantified using Bio-plex proTM mouse cytokine 23-plex assay kits (Bio-Rad #M60009RDPD). This kit detects the following concentrations of 23 analytes simultaneously in a single sample: Eotaxin (CCL11), G-CSF, GM-CSF, IFN-γ, KC, MCP-1(MCAF), MIP-1α, MIP-1β, RANTES, IL-1α, IL-1β, IL-2, IL-3, IL-4, IL-5, IL-6, IL-9, IL-10, IL-12 p40, IL-12 p70, IL-13, IL-17A and TNF-α. Prepared plates were run on the Bio-Plex 200 System with the High Throughput Fluidics (HTF) Multiplex Array System (Bio-Rad Laboratories, Hercules, CA). Results were normalized to the amount of protein per well, as determined using a Bio-Rad DC protein assay.

### Protein separation by 1D SDS-PAGE and proteomics analysis

For proteomics analysis, proteins from 7d, 1 m and 12 m groups were processed as previously described^19^. Equal amount of proteins from untreated- and treated-samples (about 60 μg) were separated by 1D SDS-PAGE, respectively. The gel bands of interest were excised from the gel, reduced with 25 mM of DTT and alkylated with 55 mM iodoacetamide. In gel digestion was then carried out with sequencing grade modified trypsin in 50 mM disodium hydrogen phosphate at 37 °C overnight. The peptides were extracted twice with 0.1% trifluoroacetic acid in 50% acetonitrile aqueous solution for 30 min. Extracts were then centrifuged in a speedvac to reduce the volume.

Peptides from different samples were labeled with tandem mass tags (TMT) reagents (Thermo, Pierce Biotechnology) according to the manufacturer’s instruction. Briefly, the TMT label reagents were dissolved by anhydrous acetonitrile and carefully added to each digestion products. The reaction was performed for 1 h at room temperature, and hydroxylamine was used to quench the reaction. The TMT-labeled peptides were desalted using the stage tips.

For LC-MS/MS analysis, the digestion product was separated by a 65 min gradient elution at a flow rate 0.250 ml/min with an EASY-nLCIITM integrated nano-HPLC system (Proxeon, Denmark) which was directly interfaced with a Thermo Orbitrap Q Executive mass spectrometer. The analytical column was a homemade fused silica capillary column (75 mm ID, 150 mm length; Upchurch, Oak Harbor, WA) packed with C-18 resin (300 Å, 5 mm, Varian, Lexington, MA). Mobile phase A consisted of 0.1% formic acid, and mobile phase B consisted of 100% acetonitrile and 0.1% formic acid. The Q Exactive mass spectrometer was operated in the data-dependent acquisition mode using Xcalibur 2.1.2 software and there was a single full-scan mass spectrum in the orbitrap (400–1800 m/z, 60,000 resolution) followed by 10 data-dependent MS/MS scans at 27% normalized collision energy (HCD). The MS/MS spectra from each LC-MS/MS run were searched against the selected database using an in house Proteome Discoverer searching algorithm.

The MS/MS spectra from each LC-MS/MS run were searched against the selected database (IPI human v3.84) using an in-house Proteome Discoverer 1.3 software (Thermo, USA). The search criteria were as follows: full tryptic specificity was required; one missed cleavage was allowed; carbamidomethylation (C) and TMT sixplex (K and N-terminal) were set as the fixed modifications; the oxidation (M) was set as the variable modification; precursor ion mass tolerances were set at 10 ppm for all MS acquired in an orbitrap mass analyzer; and the fragment ion mass tolerance was set at 20 mmu for all MS2 spectra acquired. Relative protein quantification was also performed using Proteome Discoverer software (version 1.3) according to manufacturer’s instructions on the six reporter ion intensities per peptide. Quantitative precision was expressed as protein ratio variability. Differentially expressed proteins were further confirmed by QRT-PCR or western blotting.

### Protein pathway analysis

GO annotation of the identified proteins was done using DAVID (V6.7: http://david.abcc.ncifcrf.gov). Differentially expressed proteins were analyzed using IPA (Ingenuity Systems: http:// www. ingenuity. com). The over-represented biological functions, molecular networks, and canonical pathways were generated based on information in the Ingenuity Pathways Knowledge Base.

### Statistical analysis

Data were analyzed by one-way ANOVA and post-hoc comparison tests, with significance measured at *P < 0.05; **P < 0.01.

## Results

### Establishment and characterization of primary NSC culture from different aged mice

**Figure 1:**
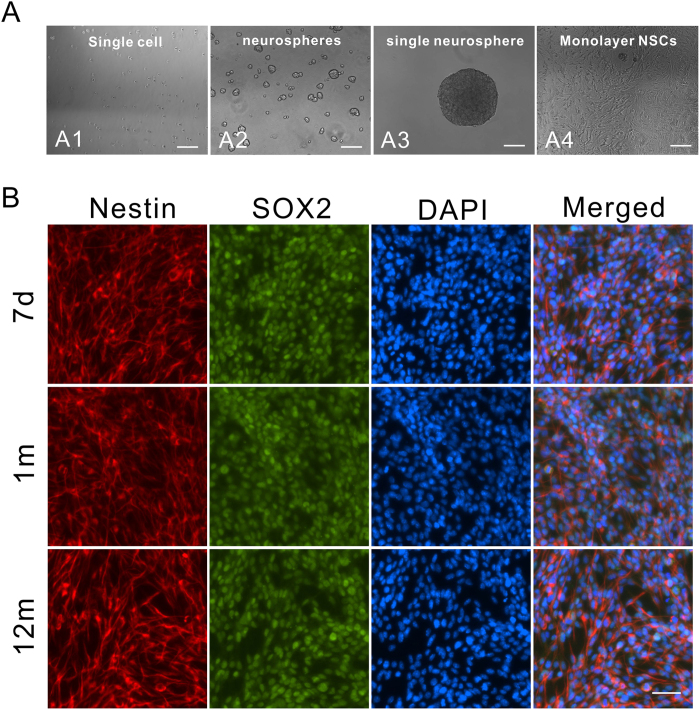
Establishment and Characterization of primary NSC culture from 7 d, 1 m, and 12 m mice. (**A**) NSCs from SVZ were cultured in the presence of EGF and bFGF. Scale bar: 100 μm. (A1) shows single cell after passaging *in vitro*. (A2) shows neurospheres. (A3) shows a single neurosphere. (A4) shows monolayer of NSCs culture on polyornithine/laminin-coated plates. (**B**) 7 d, 1 m, and 12 m neural stem cells were stained with NSC-specific markers Nestin and SOX2. Scale bar: 50 μm.

NSCs from the SVZ of postnatal 7 d mice were grown in the presence of basic fibroblast growth factor (bFGF) and epidermal growth factor (EGF) to form neurospheres *in vitro* (Fig. 1A). During subculture, the NSC from SVZ of 7 d, 1 m, and 12 m retained their stem cell characteristics and stained positively for Nestin and SOX2 (Fig. 1B).

### Changes of NSC pools in the SVZ from different aged mice

**Figure 2:**
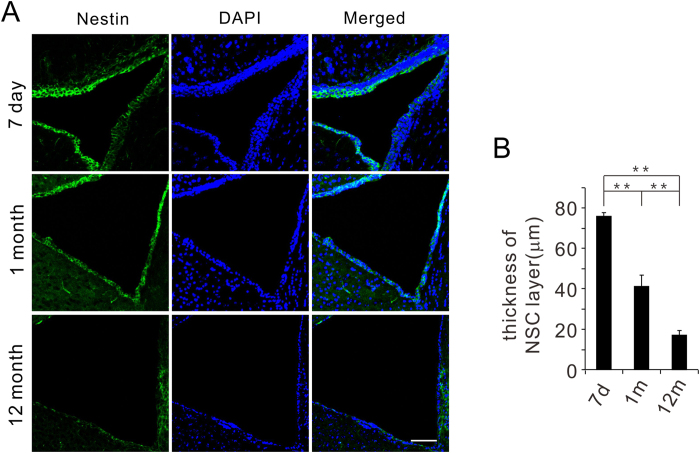
NSC pool size in the SVZ from 7 d, 1 m, 12 m mice. (**A**) Nestin immunofluorescence of the SVZ in coronal sections from 7 d, 1 m, and 12 m mice. (**B**) The thickness of the NSC layer decreases with age. **P < 0.01. Scale bar: 100 μm.

The *in vivo* age-related alterations of NSC pools in the SVZ were examined in the current study. Brains from 7 d, 1 m, and 12 m mice were stained with the anti-Nestin antibody (Fig. 2A). The results show that the width of the neurogenic area of SVZ is reduced with age (Fig. 2B).

### Neural stem cells show cellular senescence with age

**Figure 3:**
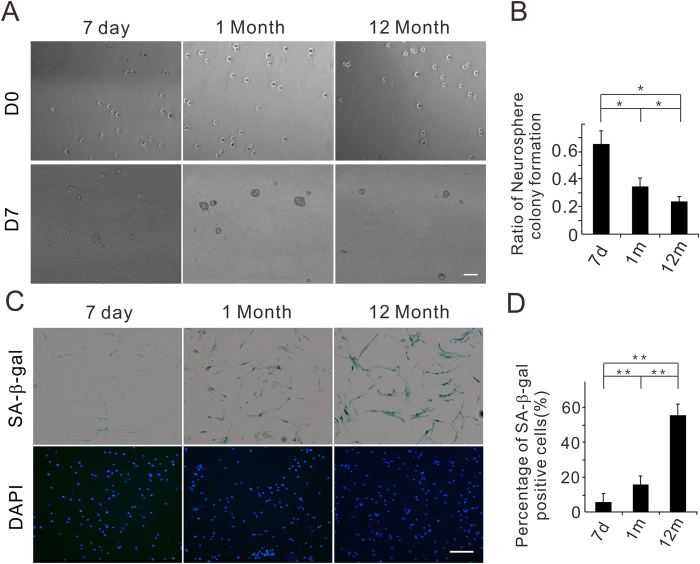
NSCs have decreased colony formation capacity and increased SA-β-gal activity with age. (**A**) Colony formation capacity of 7 d, 1 m, and 12 m NSCs. Neurospheres were counted 7 d (D7) after NSCs were plated (D0). Scale bar, 100 μm. (**B**) Quantification of the ratio of neurosphere colony formation from 7 d, 1 m to 12 m/old mice. *P < 0.05. (**C**) Senescence-associated-β-Galactosidase (SA-β-Gal) staining of 7 d, 1 m, and 12 m NSCs, showing positive cells (blue). Scale bar, 100 μm. (**D**) The ratio of SA-β-Gal positive cells from 7 d, 1 m to 12 m old mice. **P < 0.01.

To determine the proliferative capacity of isolated 7 d, 1 m, 12 m NSCs, neurosphere formation assays were carried out (Fig. 3A). The results revealed that the ratio of neurosphere formation decreased with increasing age (Fig. 3B). Next, we performed senescence-associated- β-galactosidase (SA-β-gal) assay to confirm that the NSCs aged with increasing age of the animal (Fig. 3C). The percentage of SA-β-gal- positive NSCs increased from 6.12% in 7 d to 54.31% in 12 m (Fig. 3D).

### Changes in secretome profiling of inflammatory cytokines and chemokines

**Figure 4:**
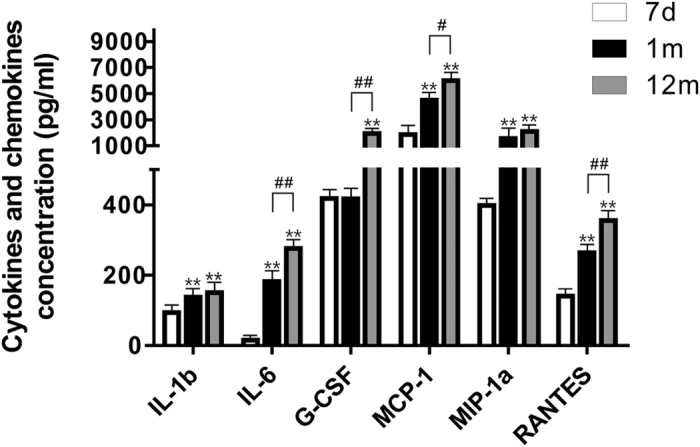
Changes in the expression levels of inflammatory cytokines and chemokines from 7 d, 1 m, and 12 m NSCs. Multiplex quantification of inflammatory chemokines and cytokines from 7 d, 1 m, and 12 m NSCs using the Bio-Plex Pro™ mouse cytokine 23-plex immunoassays. Mean ± SEM values are reported. The cytokines and chemokines not listed in the table were unchanged or not detectable. **P < 0.01 vs. 7 d; ^#^*P* < 0.05, ^##^*P* < 0.01 vs. the group indicated.

By using a Bio-rad Bio-plex pro mouse cytokine 23-plex assay kit, we characterized the changes in the expression levels of some important chemokines and cytokines released from the SVZ NSCs in 7 d, 1 m, and 12 m mice. The data revealed that a subset of molecules, such as IL-6, G-CSF, MCP-1, MIP-1a, and RANTES showed a distinct increase in expression with age. The secreted IL-6 was 18-fold from 12 m NSCs (275.96 pg/mL) compared with 7 d cells (15.3 pg/mL). The secreted MIP-1a was 5-fold from 12 m NSCs (2110.15 pg/mL) compared with 7 d cells (397.95 pg/mL). The secretion of G-CSF is elevated to nearly 5-fold (from 417.49 pg/mL to 1943.53 pg/mL). The secretion of Mcp-1 was 3-fold from 12 m NSCs (6000 pg/mL) compared with 7 d cells (1870.48 pg/mL). The secreted RANTES was 2.5-fold from 12 m NSCs (355.15 pg/mL) compared with 7 d cells (140.98 pg/mL). The data suggested that these inflammatory cytokines and chemokines may play a vital role in the aging process (Fig. 4).

### Proteomic analysis unravels major molecular pathways related to NSC aging

In order to explore the mechanisms underlying the NSC aging-related functional declines, we utilized quantitative proteomic analysis to identify the key proteins and pathways derived from neural stem cells from SVZ of 7 d, 1 m, and 12 m aged mice. Our analysis predicted 1170 distinct proteins. With a 1.5-fold cutoff, 39 proteins underwent a decline in the expression level with age while 8 proteins showed an elevation in the expression level.

**Figure 5:**
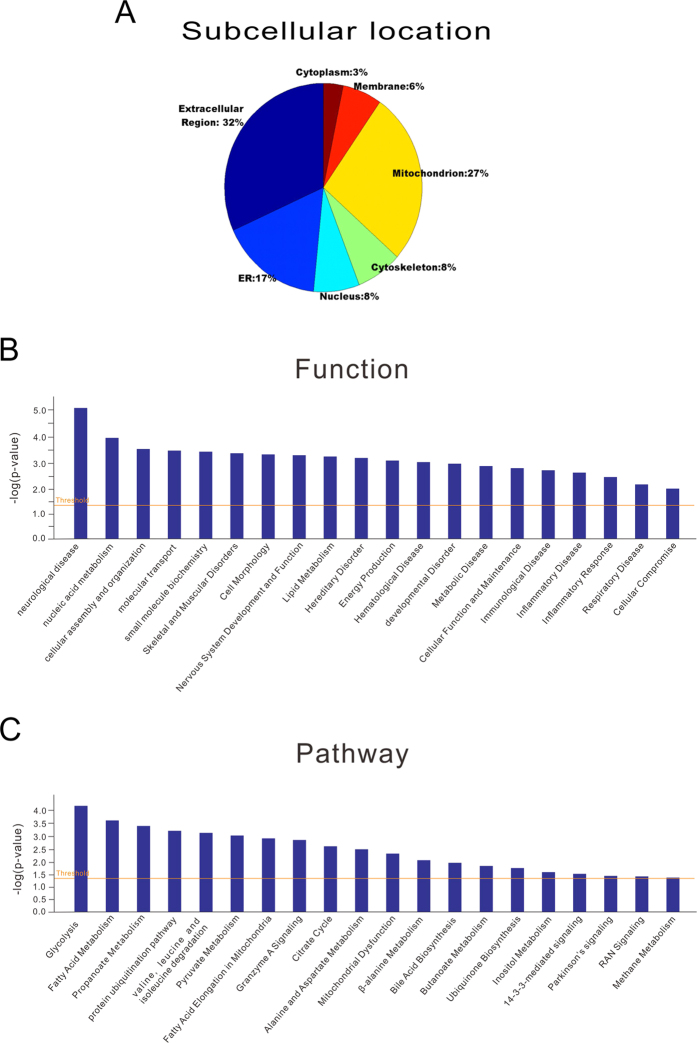
Biological function analysis of the differentially expressed proteins from 7 d, 1 m, and 12 m NSCs. (**A**) The annotations of the cellular component of the differentially expressed proteins obtained by GO analysis from the DAVID database. (**B**) Functional analysis of the significant and differentially expressed proteins with neurological disease, nucleic acid metabolism, cellular assembly and organization, molecular transport, and small molecule biochemistry. (**C**) The most significant canonical pathways altered from 7 d, 1 m, and 12 m-old NSCs including glycolysis, fatty acid metabolism, propanoate metabolism, protein ubiquitination pathway, valine, leucine, and isoleucine.

Subsequently, we performed bioinformatics analysis on the differentially expressed proteins using the DAVID bioinformatics tool. The majority of the differentially expressed proteins were located in the extracellular region (32%), the mitochondrion (27%), and the endoplasmic reticulum (17%) (Fig. 5A). Functionally these genes were mainly associated with the neurological disease, nucleic acid metabolism, cellular assembly and organization, molecular transport, and small molecule biochemistry (Fig. 5B). The main 5 canonical pathways highly influenced were related to glycolysis, fatty acid metabolism, propanoate metabolism, protein ubiquitination pathway, and valine, leucine, and isoleucine degradation (Fig. 5C).

**Figure 6:**
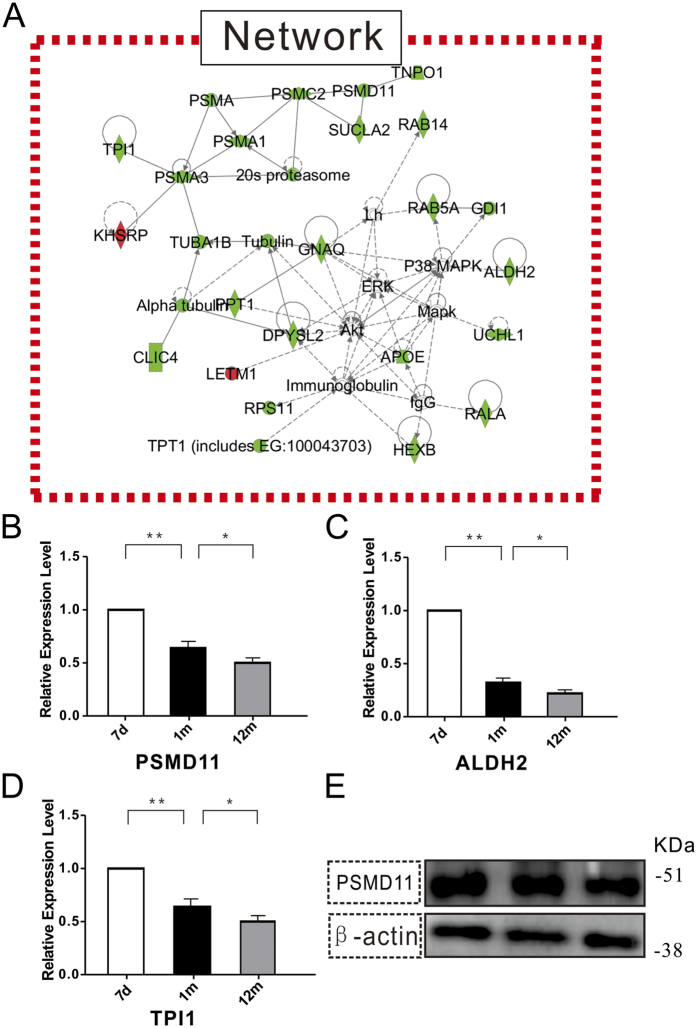
Candidate genes and pathways identified through the 7 d, 1 m, and 12 m NSCs associated with the loss of proteostasis and neurodegenerative disease. (**A**) The key regulatory networks underlying the aging-associated NSCs. The proteomic data was imported into the Ingenuity Pathway Analysis (IPA) to predict the interacting pathways. Green indicates a decrease in the protein expression and red represents an increase. The color intensity represents the alteration in the level of expression change; the solid and dashed lines indicate the direct and indirect interactions, respectively, on the pathways. The network with the highest network score of 68 was associated with neurological disease, lipid metabolism, and small molecule biochemistry. (**B**,**C**,**D**) PSMD11, ALDH2, and TPI1 expression was confirmed by qRT-PCR using GAPDH as an internal control. The data are presented as mean ± SEM from three independent experiments. (**E**) PSMD11 protein expression was confirmed by Western blot with β-actin as an internal control.

Next, we used the Ingenuity Pathway Analysis (IPA) pathway to identify major cellular functional networks altered in age-associated NSCs. 24 proteins were involved in the top-hit network, with the highest score of 63. Functionally, these proteins were mainly associated with neurological disease, lipid metabolism, and small molecule biochemistry. Importantly, the downregulated expression patterns of these proteins are highly correlated with the loss of proteostasis and neurodegenerative diseases such as Parkinson’s and Alzheimer’s diseases. For example, the proteasome subunit alpha type-1 (PSMA1), proteasome subunit alpha type-3 (PSMA3), PSMC2, PSMD11, and UCHL1 were decreased with age, which was consistent with the loss of proteostasis and NSC senescence. On the other hand, TPI1, ALDH2, DPYSL2, and ApoE were decreased with age, consistent with the involvement in the neurodegeneration processes (Fig. 6A).

Furthermore, we validated the proteomic data by a qRT-PCR and Western blot. As shown in Fig. 6C,D, the relative expression levels of PSMD11, TPI1, and ALDH2 mRNA were decreased, according to qRT-PCR (Fig. 6B–D) while that of PSMD11 was decreased as confirmed by Western blot. These results were in complete agreement with the quantitative proteomic results (Fig. 6E).

## Discussion

In the present study, we have shown that the width of the SVZ and the pool size of NSCs, is reduced from 7 d, 1 m to 12 m. We also observed a significant reduction in the level of NSC proliferation with age as demonstrated by neurosphere formation assays. These pieces of evidence suggest that NSCs are directed towards cell senescence with age, which is also determined by augmented SA-β-gal staining.

Furthermore, our results demonstrated that several critical inflammatory cytokines and chemokines such as IL-6, G-CSF, MCP-1, and RANTES are significantly increased with age. This prominent aging-associated alteration is “inflammaging”^20^,^21^, which is tightly associated with age-related diseases, such as Alzheimer’s disease, Parkinson’s disease, atherosclerosis, and heart diseases. In the brain, the aged neurogenic niche is characterized by elevated levels of inflammatory cytokines. This increased inflammation contributes to the declining function of NSCs. One of the most reliable biomarkers of aging is IL-6, which is a potent mediator of inflammatory processes^22^. Herein, we found that the secretion of IL-6 is elevated to 18-fold in 7 d NSCs as compared to 12 m, which suggested that IL-6 levels can be used as predictors of inflammaging in NSC. MCP-1 is a vital chemokine; Inadera *et al*. measured the MCP-1 levels, using ELISA, in 405 healthy Japanese subjects of various ages, and found that the increase was age-dependent^23^. Chiao *et al*. measured the levels of 69 analytes in the plasma of adult and senescent C57/BL6 mice by multi-analyte profiling, and the results suggested that MCP-1 may serve as a potential plasma marker for cardiac aging^23^. Felzien *et al*. found another C-C chemokines, normally expressed in T-cell and secreted RANTES, and their level increase in T-cells, midbrain, hippocampus, and cerebellum with age^24^.

In order to understand the underlying mechanisms modulating the age-related changes and protein profiles of NSCs in the SVZ, and to predict the neurodegenerative diseases, we utilized quantitative proteomics analysis. We focused on several proteins that showed significant expression changes, designating them as potential NSC senescence markers. Interestingly, PSMA1, which is a core alpha subunit of the 20 S proteasome^25^,^26^, showed a decreased expression level with aging. The specific inhibition of the 20 S proteasome led to accelerated cell senescence^27^,^28^,^29^,^30^, hence, PSMA1 could be used as a novel senescence-associated marker of NSCs. Another molecule, PSMD11 also decreased with NSC aging. As is known, embryonic stem cells can replicate continuously in the absence of senescence, which can be explained by the increased levels of PSMD11^31^.

Ingenuity Pathway Analysis was used to investigate the potential changes in the molecular functions and group proteins into several highly possible networks. Through IPA, our quantitative proteomic results revealed changes in some proteins that play critical roles in protein homeostasis. Several proteins, including PSMA1, PSMA3, PSMC2, PSMD11, UCHL1 in the network 1 showing significant downregulation have been reported to be directly correlated to ubiquitin-proteasome system. Also, protein ubiquitination pathway is one of the top five enriched canonical pathways according to our bioinformatics analysis on the differentially expressed proteins as predicted by the DAVID Bioinformatics Resource. The ubiquitin-proteasome system is the primary source of intracellular protein degradation, involved in more than 80% protein degradation in the cell.

Proteome homeostasis plays a critical role in the health of the cell^32^, and its loss is a major hallmark of aging^1^. With age, misfolded, or damaged proteins have detrimental consequences on protein homeostasis, leading to the development of several age-related pathologies^33^,^34^. In the present study, UCHL1 was found to be downregulated with age. It is a core protein in the ubiquitin/proteasome pathway, which is responsible for the degradation of intracellular proteins and distinctly misfolded and damaged proteins. It is well-known that the hallmark of the Parkinson’s disease is the widespread occurrence of α-synuclein-positive Lewy bodies and dystrophic Lewy neurites throughout the brain. The inhibition of UCHL1 is associated with the production of α-Syn aggregates, which can lead to PD^35^.

Through IPA analysis, we found 24 proteins involved in the neurological disease, lipid metabolism, and small molecule biochemistry that were grouped as the top network (Fig. 6). Among them, several proteins were associated with neurodegenerative diseases, and all these were found to be downregulated with age. DPYSL2 plays a role in neuronal development, neural differentiation, neurotransmitter release and polarity, as well as in axonal growth and guidance, neuronal growth cone collapse, and cell migration. This gene has been further implicated in multiple neurological disorders, and hyperphosphorylation of the encoded protein may play a vital role in the development of AD as the protein level was decreased in AD patients.

Triosephosphate isomerase 1 (TPI1) is a glycolytic enzyme, essential in the glycolytic pathway. It can cause an accumulation of methylglyoxal and subsequent increase in the generation of advanced glycation end products (AGEs), and its activities were found to be significantly reduced in AD patients.

Aldehyde dehydrogenase (ALDH) is the second enzyme of the major oxidative pathway of alcohol metabolism. It metabolizes acetaldehyde into acetate and protects against oxidative stress. Several pieces of evidence suggest that ALDH plays a significant role in the development of AD^36^,^37^, and its protein expression and activity are substantially decreased in the substantia nigra of patients with PD.

In summary, we found that the pool size and the colony formation capacity of NSCs decreased and cell senescence increased from 7 d, 1 m to 12 m, and the cellular senescence triggers senescence-associated inflammatory cytokine secretion. Next, we utilized quantitative proteomic analysis to estimate the regulation of 1170 distinct proteins derived from NSCs in the SVZ of 7 d, 1 m, and 12 m-old mice. With a 1.5-fold cutoff, 47 distinct proteins were significantly altered. Our proteomic data revealed that protein ubiquitination pathway lost its normal function during the NSC aging process. PSMD11 was preliminarily validated by Western blot in our study and emerged a promising marker of aging in NSCs. Several proteins were associated with neurodegenerative diseases, DPYSL2 and TPI1 were related to AD, UCHL1 was linked to PD, whereas ALDH was related to AD and PD. Our data show that impaired proteostasis is one of the important hallmarks of brain aging, which contributes to progressive neurodegenerative diseases. This will provide an insight into the potential pathways and regulatory networks involved in NSC aging which will help to identify drug targets for improving human longevity and predict neurodegenerative diseases at an early stage.

## Additional Information

**How to cite this article**: Wang, X. *et al*. Quantitative proteomic analysis of age-related subventricular zone proteins associated with neurodegenerative disease. *Sci. Rep.* **6**, 37443; doi: 10.1038/srep37443 (2016).

**Publisher's note**: Springer Nature remains neutral with regard to jurisdictional claims in published maps and institutional affiliations.

## References

[b1] Lopez-Otin C., Blasco M. A., Partridge L., Serrano M. & Kroemer G. The hallmarks of aging. Cell 153, 1194–1217, doi: 10.1016/j.cell.2013.05.039 (2013). 23746838 PMC3836174

[b2] Liu L. & Rando T. A. Manifestations and mechanisms of stem cell aging. J Cell Biol 193, 257–266, doi: 10.1083/jcb.201010131 (2011). 21502357 PMC3080271

[b3] Della-Maggiore V., Grady C. L. & McIntosh A. R. Dissecting the effect of aging on the neural substrates of memory: deterioration, preservation or functional reorganization? Reviews in the neurosciences 13, 167–181 (2002). 12160260 10.1515/revneuro.2002.13.2.167

[b4] Winner B., Kohl Z. & Gage F. H. Neurodegenerative disease and adult neurogenesis. The European journal of neuroscience 33, 1139–1151, doi: 10.1111/j.1460-9568.2011.07613.x (2011). 21395858

[b5] Signer R. A. & Morrison S. J. Mechanisms that regulate stem cell aging and life span. Cell stem cell 12, 152–165, doi: 10.1016/j.stem.2013.01.001 (2013). 23395443 PMC3641677

[b6] Ming G. L. & Song H. Adult neurogenesis in the mammalian brain: significant answers and significant questions. Neuron 70, 687–702, doi: 10.1016/j.neuron.2011.05.001 (2011). 21609825 PMC3106107

[b7] Doetsch F., Caille I., Lim D. A., Garcia-Verdugo J. M. & Alvarez-Buylla A. Subventricular zone astrocytes are neural stem cells in the adult mammalian brain. Cell 97, 703–716 (1999). 10380923 10.1016/s0092-8674(00)80783-7

[b8] Maslov A. Y., Barone T. A., Plunkett R. J. & Pruitt S. C. Neural stem cell detection, characterization, and age-related changes in the subventricular zone of mice. The Journal of neuroscience: the official journal of the Society for Neuroscience 24, 1726–1733, doi: 10.1523/jneurosci.4608-03.2004 (2004). 14973255 PMC6730468

[b9] Molofsky A. V. . Increasing p16INK4a expression decreases forebrain progenitors and neurogenesis during ageing. Nature 443, 448–452, doi: 10.1038/nature05091 (2006). 16957738 PMC2586960

[b10] Katyal S. & McKinnon P. J. DNA strand breaks, neurodegeneration and aging in the brain. Mechanisms of ageing and development 129, 483–491, doi: 10.1016/j.mad.2008.03.008 (2008). 18455751 PMC3831510

[b11] Savitt J. M., Dawson V. L. & Dawson T. M. Diagnosis and treatment of Parkinson disease: molecules to medicine. The Journal of clinical investigation 116, 1744–1754, doi: 10.1172/jci29178 (2006). 16823471 PMC1483178

[b12] van den Berge S. A., van Strien M. E. & Hol E. M. Resident adult neural stem cells in Parkinson’s disease-the brain’s own repair system? European journal of pharmacology 719, 117–127, doi: 10.1016/j.ejphar.2013.04.058 (2013). 23872414

[b13] Young S. Z., Taylor M. M. & Bordey A. Neurotransmitters couple brain activity to subventricular zone neurogenesis. The European journal of neuroscience 33, 1123–1132, doi: 10.1111/j.1460-9568.2011.07611.x (2011). 21395856 PMC3075963

[b14] Ziabreva I. . Altered neurogenesis in Alzheimer’s disease. Journal of psychosomatic research 61, 311–316, doi: 10.1016/j.jpsychores.2006.07.017 (2006). 16938507

[b15] Seaberg R. M. & van der Kooy D. Adult rodent neurogenic regions: the ventricular subependyma contains neural stem cells, but the dentate gyrus contains restricted progenitors. The Journal of neuroscience: the official journal of the Society for Neuroscience 22, 1784–1793 (2002). 11880507 10.1523/JNEUROSCI.22-05-01784.2002 PMC6758891

[b16] Rodriguez J. J. . Impaired adult neurogenesis in the dentate gyrus of a triple transgenic mouse model of Alzheimer’s disease. PloS one 3, e2935, doi: 10.1371/journal.pone.0002935 (2008). 18698410 PMC2492828

[b17] Rodriguez J. J., Jones V. C. & Verkhratsky A. Impaired cell proliferation in the subventricular zone in an Alzheimer’s disease model. Neuroreport 20, 907–912, doi: 10.1097/WNR.0b013e32832be77d (2009). 19494789

[b18] Dimri G. P. . A biomarker that identifies senescent human cells in culture and in aging skin *in vivo*. Proceedings of the National Academy of Sciences of the United States of America 92, 9363–9367 (1995). 7568133 10.1073/pnas.92.20.9363 PMC40985

[b19] Dong C. M. . A stress-induced cellular aging model with postnatal neural stem cells. Cell Death Dis 5, e1116, doi: 10.1038/cddis.2014.82 (2014). 24625975 PMC3973228

[b20] De la Fuente M. & Miquel J. An update of the oxidation-inflammation theory of aging: the involvement of the immune system in oxi-inflamm-aging. Current pharmaceutical design 15, 3003–3026 (2009). 19754376 10.2174/138161209789058110

[b21] Franceschi C. . Inflammaging and anti-inflammaging: a systemic perspective on aging and longevity emerged from studies in humans. Mechanisms of ageing and development 128, 92–105, doi: 10.1016/j.mad.2006.11.016 (2007). 17116321

[b22] Campisi J. Aging, cellular senescence, and cancer. Annu Rev Physiol 75, 685–705, doi: 10.1146/annurev-physiol-030212-183653 (2013). 23140366 PMC4166529

[b23] Inadera H., Egashira K., Takemoto M., Ouchi Y. & Matsushima K. Increase in circulating levels of monocyte chemoattractant protein-1 with aging. Journal of interferon & cytokine research: the official journal of the International Society for Interferon and Cytokine Research 19, 1179–1182, doi: 10.1089/107999099313127 (1999). 10547158

[b24] Felzien L. K., McDonald J. T., Gleason S. M., Berman N. E. & Klein R. M. Increased chemokine gene expression during aging in the murine brain. Brain research 890, 137–146 (2001). 11164776 10.1016/s0006-8993(00)03090-0

[b25] Chondrogianni N., Fragoulis E. G. & Gonos E. S. Protein degradation during aging: the lysosome-, the calpain- and the proteasome-dependent cellular proteolytic systems. Biogerontology 3, 121–123 (2002). 12014830 10.1023/a:1015236203379

[b26] Davies K. J. Degradation of oxidized proteins by the 20S proteasome. Biochimie 83, 301–310 (2001). 11295490 10.1016/s0300-9084(01)01250-0

[b27] Awasthi N. & Wagner B. J. Suppression of human lens epithelial cell proliferation by proteasome inhibition, a potential defense against posterior capsular opacification. Investigative ophthalmology & visual science 47, 4482–4489, doi: 10.1167/iovs.06-0139 (2006). 17003443

[b28] Chondrogianni N. & Gonos E. S. Proteasome inhibition induces a senescence-like phenotype in primary human fibroblasts cultures. Biogerontology 5, 55–61 (2004). 15138382 10.1023/b:bgen.0000017687.55667.42

[b29] Chondrogianni N. . Central role of the proteasome in senescence and survival of human fibroblasts: induction of a senescence-like phenotype upon its inhibition and resistance to stress upon its activation. The Journal of biological chemistry 278, 28026–28037, doi: 10.1074/jbc.M301048200 (2003). 12736271

[b30] Stapnes C. . The proteasome inhibitors bortezomib and PR-171 have antiproliferative and proapoptotic effects on primary human acute myeloid leukaemia cells. British journal of haematology 136, 814–828, doi: 10.1111/j.1365-2141.2007.06504.x (2007). 17341267

[b31] Vilchez D. . Increased proteasome activity in human embryonic stem cells is regulated by PSMD11. Nature 489, 304–308, doi: 10.1038/nature11468 (2012). 22972301 PMC5215918

[b32] Powers E. T., Morimoto R. I., Dillin A., Kelly J. W. & Balch W. E. Biological and chemical approaches to diseases of proteostasis deficiency. Annual review of biochemistry 78, 959–991, doi: 10.1146/annurev.biochem.052308.114844 (2009). 19298183

[b33] Koga H., Kaushik S. & Cuervo A. M. Protein homeostasis and aging: The importance of exquisite quality control. Ageing research reviews 10, 205–215, doi: 10.1016/j.arr.2010.02.001 (2011). 20152936 PMC2888802

[b34] Zhang C. & Cuervo A. M. Restoration of chaperone-mediated autophagy in aging liver improves cellular maintenance and hepatic function. Nature medicine 14, 959–965, doi: 10.1038/nm.1851 (2008). PMC2722716 18690243

[b35] Healy D. G., Abou-Sleiman P. M. & Wood N. W. Genetic causes of Parkinson’s disease: UCHL-1. Cell and tissue research 318, 189–194, doi: 10.1007/s00441-004-0917-3 (2004). 15221445

[b36] Ohta S. & Ohsawa I. Dysfunction of mitochondria and oxidative stress in the pathogenesis of Alzheimer’s disease: on defects in the cytochrome c oxidase complex and aldehyde detoxification. Journal of Alzheimer’s disease: JAD 9, 155–166 (2006). 10.3233/jad-2006-9208 16873963

[b37] Markesbery W. R. The role of oxidative stress in Alzheimer disease. Archives of neurology 56, 1449–1452 (1999). 10593298 10.1001/archneur.56.12.1449

